# A New Analytic Alignment Method for a SINS

**DOI:** 10.3390/s151127930

**Published:** 2015-11-04

**Authors:** Caiming Tan, Xinhua Zhu, Yan Su, Yu Wang, Zhiqiang Wu, Dongbing Gu

**Affiliations:** 1 School of Mechanical Engineering, Nanjing University of Science and Technology, Nanjing 210094, China; E-Mails: tancm314@hotmail.com (C.T.); suyan@mail.njust.edu.cn (Y.S.); wangyu.njust@gmail.com (Y.W.); wuzhiqiang@mail.njust.edu.cn (Z.W.); 2 School of Computer Science and Electronic Engineering, University of Essex, Essex CO4 3SQ, UK; E-Mail: dgu@essex.ac.uk

**Keywords:** SINS, self-alignment, initial alignment, analytic alignment, TRIAD

## Abstract

Analytic alignment is a type of self-alignment for a Strapdown inertial navigation system (SINS) that is based solely on two non-collinear vectors, which are the gravity and rotational velocity vectors of the Earth at a stationary base on the ground. The attitude of the SINS with respect to the Earth can be obtained directly using the TRIAD algorithm given two vector measurements. For a traditional analytic coarse alignment, all six outputs from the inertial measurement unit (IMU) are used to compute the attitude. In this study, a novel analytic alignment method called selective alignment is presented. This method uses only three outputs of the IMU and a few properties from the remaining outputs such as the sign and the approximate value to calculate the attitude. Simulations and experimental results demonstrate the validity of this method, and the precision of yaw is improved using the selective alignment method compared to the traditional analytic coarse alignment method in the vehicle experiment. The selective alignment principle provides an accurate relationship between the outputs and the attitude of the SINS relative to the Earth for a stationary base, and it is an extension of the TRIAD algorithm. The selective alignment approach has potential uses in applications such as self-alignment, fault detection, and self-calibration.

## 1. Introduction

If the Strapdown inertial navigation system (SINS) remains static on the ground with limited vibrations, the attitude of the SINS with respect to the Earth can be obtained directly from the gravity and rotational velocity vectors of the Earth if they are not collinear [1,2,3,4]. This approach is called analytic alignment (AA). The problem is about finding the transformation matrix from one coordinate system to the other when the components of two abstract vectors are given in two different coordinate systems. TRIAD [5,6] is an algorithm that does just that. A minimum of two non-coplanar vector pairs are required for a solution using TRIAD algorithm. The TRIAD algorithm is applied to the AA for a SINS, and then it is called the analytic coarse alignment method [1,2,3,4]. If the vibrations are so severe that the outputs of the gyroscopes are much larger than the rotational velocity of the Earth, AA cannot be performed. There are many approaches to the alignment problem including the inertial frame alignment method [7,8], Kalman fine alignment [9,10,11], and compass alignment [12,13]. All of these methods require part or all of the inertial navigation process, where the velocity and attitude of the SINS update with the time. Because the average velocity of the SINS is at zero for the base in the presence of vibrations, the attitude could then be computed. These alignment methods for the base in the presence of vibrations can be classified as inertial navigation computational alignment (INCA). However, INCA requires more alignment time and involves massive calculation compared to the AA method. Self-alignment methods can be divided into AA and INCA based on the principles used.

Because vibration is common in actual systems, INCA has been widely studied for several decades [7,8,9,10,12]. There have been few studies on AA, mainly because it has difficulty achieving alignment in the presence of vibrations. Reference [1] presents a basic AA method, analytic coarse alignment. One additional vector can be generated with the vector cross product between the gravity and rotational velocity vectors of the Earth. Then, a 3 × 3 matrix can be formed given the three vectors. In a SINS, the attitude information is stored either as a coordinate transformation matrix or as attitude angles (roll, pitch and yaw). The coordinate transformation matrix can be obtained directly through a one-time matrix calculation. Jiang [2] noted that there are three other vectors that can be generated from the cross-product of the gravity and rotational velocity vectors. Among these five vectors, four lie in the plane of the meridian and the other is perpendicular to this plane. The analytic coarse alignment problem is then to form a 3 × 3 matrix that consists of three linearly independent vectors. There are six possible sets that can be used to build a 3 × 3 matrix for the analytic coarse alignment. Two simple and significant sets are analyzed in [2] in detail. Although both methods are derived from the same measurements of the local gravity and rotational velocity vectors, their error formulations are not completely identical. The six analytic coarse alignment methods and a direct method for a SINS on stationary base were compared using error analyses in [3]. The error characteristics of the direct method can be evaluated because the direct method is equivalent to one of the six analytic coarse alignment methods.

It has been the standard practice for many years to use all six outputs from the IMU for self-alignment. A new AA method called selective alignment in ground platforms is proposed which requires only three outputs from the IMU and a few properties from the remaining outputs such as the sign and the approximate value. The selective alignment method has potential uses in problems such as optimal AA, fault detection, and self-calibration.

The selective alignment method shows that two non-coplanar vector pairs are redundant for determining the transformation matrix, however, a minimum of two non-coplanar vector pairs are required for a solution using TRIAD algorithm. The selective alignment may be applied in other attitude determination problems such as spacecraft attitude estimation where TRIAD algorithm is a basic method [6,14]. The formulas for the selective alignment are given through geometrical analysis in this paper. The principle could also be obtained through algebra analysis and we give a brief description about this in a recent conference paper [15]. After all, the geometrical analysis for the selective alignment shows us a much more intuitive and figurative relationship between the IMU outputs and its attitude relative to the Earth on the stationary base.

The remainder of the paper is organized as follows: We review the analytic coarse alignment method in Section 2. The basic principle of selective alignment is presented in Section 3. To prevent a zero value in the denominator or a negative value in the radicand at certain extreme attitudes, certain adjustments are required, the details of which are presented in Section 4. The results of simulations and experiments to test the validity and the use of the selective alignment method are presented in Section 5. Section 6 provides concluding remarks.

## 2. Analytic Coarse Alignment

The analytic coarse alignment is an application of the TRIAD algorithm to the AA for a SINS [2,6].

The process of finding the matrix using TRIAD is as follows [2,6,16]. Let $u_{1}$ and $u_{2}$ denote the column vectors whose elements are, respectively, the components of the two abstract vectors when resolved in one coordinate system (typically a body frame), and let $v_{1}$ and $v_{2}$ denote the column vectors whose elements are, respectively, the components of the abstract vectors when resolved in the other coordinate system (typically a reference frame). The reference-to-body coordinate transformation matrix *A* satisfies:

(1) $$u_{1}=Av_{1},\;u_{2}=Av_{2}$$

The algorithm calls for the computation of the following column vectors in the body frame:

(2) $$r_{1}=u_{1}/\left|u_{1}\right|,\;r_{2}=u_{2}/\left|u_{2}\right|,\;r_{3}=\left(u_{1}\times u_{2}\right)/\left|u_{1}\times u_{2}\right|,\;r_{4}=r_{3}\times r_{1},\;r_{5}=r_{3}\times r_{2}$$

and the following corresponding column vectors in the reference frame:

(3) $$s_{1}=v_{1}/\left|v_{1}\right|,\;s_{2}=v_{2}/\left|v_{2}\right|,\;s_{3}=\left(v_{1}\times v_{2}\right)/\left|v_{1}\times v_{2}\right|,\;s_{4}=s_{3}\times s_{1},\;s_{5}=s_{3}\times s_{2}$$

of which four vectors are at the same plane except $s_{3}$ which is perpendicular to the plane. The coordinate matrix *A* can be obtained by

(4) $$A=\left[r_{3},r_{i},r_{j}\right]{\left[s_{3},s_{i},s_{j}\right]}^{-1}$$

where i,j=1,2,4,5, and i<j. There are six different sets to yield A.

**Figure 1:**
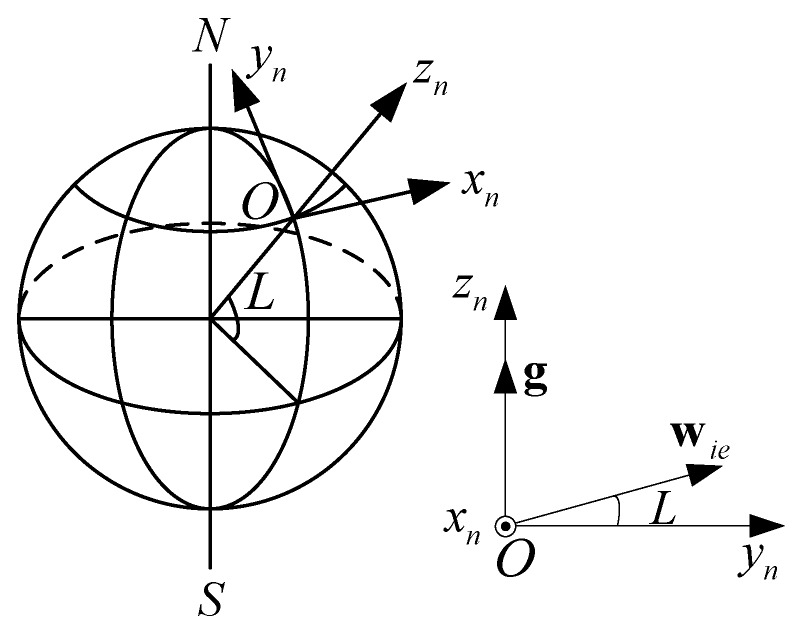
The gravity and rotational velocity vectors in the navigation frame.

When the SINS is static with respect to the Earth, there are only two inputs for the IMU: the gravity and rotational velocity vectors of the Earth, which are denoted by g and $w_{ie}$, respectively, and are shown in Figure 1. A local ENU (East-North-Up) frame is used as the reference frame, or navigation frame, denoted by $O-x_{n}y_{n}z_{n}$. The body frame of the SINS is denoted by $O-x_{b}y_{b}z_{b}$.

Neglecting sensor errors and base vibrations, the following measurements are available:

(5) $$\{\begin{array}{l} f^{b}=C_{n}^{b}g^{n}\\ w^{b}=C_{n}^{b}w_{ie}^{n} \end{array}$$

where $f^{b}$ contains the outputs of a three-axis accelerometer with $f^{b}={\left[f_{x},f_{y},f_{z}\right]}^{\text{T}}$, $w^{b}$ contains the outputs of a three-axis gyroscope with $w^{b}={\left[w_{x},w_{y},w_{z}\right]}^{\text{T}}$, $g^{n}={\left[0,0,g\right]}^{\text{T}}$ and g is the gravitational acceleration, $w_{ie}^{n}={\left[0,w_{ie}\cos L,w_{ie}\sin L\right]}^{\text{T}}$ with $w_{ie}$ and *L* denoting the rotation rate of the Earth and the latitude, respectively, and $C_{n}^{b}$ is the navigation-to-body coordinate transformation matrix. From Equations (4) and (5), the following two equations can be obtained, and these are typically used in analytic coarse alignment methods [2,3].

(6a) $$C_{n}^{b}=\left[f^{b},w^{b},f^{b}\times w^{b}\right]{\left[g^{n},w_{ie}^{n},g^{n}\times w_{ie}^{n}\right]}^{-1}$$

(6b) $$C_{n}^{b}=\left[f^{b},f^{b}\times w^{b},(f^{b}\times w^{b})\times f^{b}\right]{\left[g^{n},g^{n}\times w_{ie}^{n},(g^{n}\times w_{ie}^{n})\times g^{n}\right]}^{-1}$$

## 3. Selective Alignment

**Figure 2:**
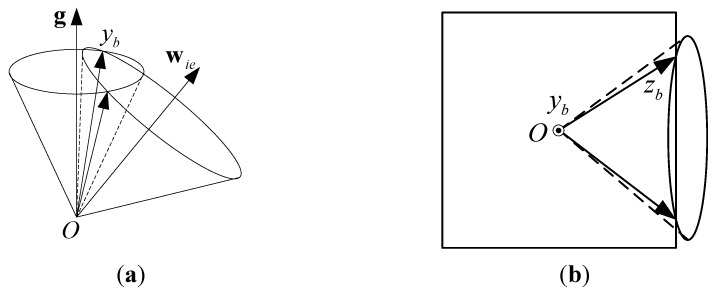
Existence of the selective alignment solution: (**a**) Two lines that represent the $y_{b}$-axis will be formed by the intersections of the two conical surfaces; (**b**) Two lines that represent the $z_{b}$-axis will be formed by the intersection of a plane and a conical surface.

If one of the IMU outputs, $f_{y}$ for example, is known, the angle between the $y_{b}$-axis and g can be computed. If $w_{y}$ is also given, the angle between the $y_{b}$-axis and $w_{ie}$ can be determined. As shown in Figure 2a, there are two possible solutions for the $y_{b}$-axis because usually there will be two lines of intersection between the two conical surfaces.

After the $y_{b}$-axis is obtained, the $O-x_{b}y_{b}z_{b}$ frame can be obtained if one more axis is computed. For example, if $w_{z}$ is also known, the $z_{b}$-axis will be on the conical surface with its center at $w_{ie}$. In addition, because the $z_{b}$-axis is normal to the $y_{b}$-axis, the $z_{b}$-axis must be in the plane that is normal to the $y_{b}$-axis. There are usually two lines of intersection of a plane and a conical surface that represent the $z_{b}$-axis, as shown in Figure 2b.

Given these facts, there are usually four possible results for the $O-x_{b}y_{b}z_{b}$ frame given $f_{y}$, $w_{y}$, and $w_{z}$.

### 3.1. Derivation 

**Figure 3:**
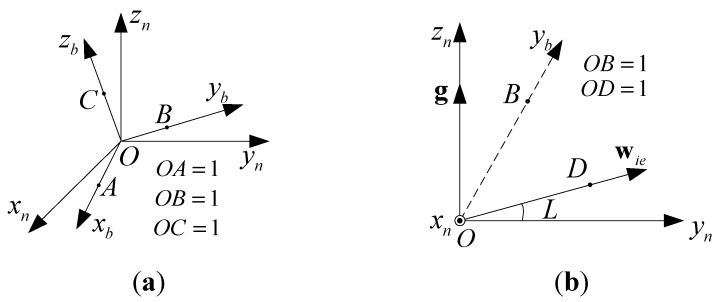
(**a**) The definitions of $\vec{OA}$, $\vec{OB}$, and $\vec{OC}$; (**b**) $\vec{OB}$, namely, the $y_{b}$-axis, can be determined given $w_{y}$ and $f_{y}$. Here, the $y_{b}$-axis is not usually in the O−yz plane.

Now let us derive mathematical expressions for the four solutions of $O-x_{b}y_{b}z_{b}$. As shown in Figure 3a, $O-x_{n}y_{n}z_{n}$ denotes the navigation coordinates, as indicated by the subscript n, and $O-x_{b}y_{b}z_{b}$ denotes the body coordinates, as indicated by the subscript b. Let $\vec{OA}=(x_{x},y_{x},z_{x})$, $\vec{OB}=(x_{y},y_{y},z_{y})$, and $\vec{OC}=(x_{z},y_{z},z_{z})$ be coordinates in the navigation frame, and let $\vec{OA}=(1,0,0)$, $\vec{OB}=(0,1,0)$, and $\vec{OC}=(0,0,1)$ be coordinates in the body frame. After ascertaining $O-x_{b}y_{b}z_{b}$, $C_{n}^{b}$ can be obtained by

(7) $$C_{n}^{b}=\left[\begin{array}{ccc} x_{x} & y_{x} & z_{x}\\ x_{y} & y_{y} & z_{y}\\ x_{z} & y_{z} & z_{z} \end{array}\right]$$

Assume three outputs of the IMU are given, which are $f_{y}$, $w_{y}$, and $w_{z}$. If $w_{y}$ is given, as shown in Figure 3b, we have

(8) $$\cos∠BOD=\frac{w_{y}}{w_{ie}}$$

Referring to Figure 3b, in the navigation frame the following equation holds:

(9) $$\vec{OB}\cdot \vec{OD}=OB\cdot OD\cdot \cos∠BOD$$

Because $\vec{OB}$ and $\vec{OD}$ are unit vectors and $\vec{OD}=\frac{w_{ie}^{n}}{w_{ie}}$, substituting Equation (8) into Equation (9) gives

(10) $$y_{y}\cos L+z_{y}\sin L=\frac{w_{y}}{w_{ie}}$$

If $f_{y}$ is given, the angle between $\vec{OB}$ and the z-axis is known, which implies

(11) $$z_{y}=\frac{f_{y}}{g}$$

Combining Equations (10) and (11), and OB=1 yields

(12) $$\{\begin{array}{l} y_{y}\cos L+z_{y}\sin L=\frac{w_{y}}{w_{ie}}\\ z_{y}=\frac{f_{y}}{g}\\ \sqrt{x_{y}^{2}+y_{y}^{2}+z_{y}^{2}}=1 \end{array}$$

Solving Equation (12) yields two sets of solutions:

(13) $$\{\begin{array}{l} x_{y}=\pm \sqrt{1-y_{y}^{2}-z_{y}^{2}}\\ y_{y}=\frac{w_{y}}{w_{ie}\cos L}-z_{y}\tan L\\ z_{y}=\frac{f_{y}}{g} \end{array}$$

Similarly, if $w_{z}$ is given, we have

(14) $$y_{z}\cos L+z_{z}\sin L=\frac{w_{z}}{w_{ie}}$$

As shown in Figure 3a, because $\vec{OC}$ is the unit vector of the positive $z_{b}$-axis and $\vec{OC}⊥\vec{OB}$, the following equations hold

(15) $$\{\begin{array}{l} \sqrt{x_{z}^{2}+y_{z}^{2}+z_{z}^{2}}=1\\ x_{y}x_{z}+y_{y}y_{z}+z_{y}z_{z}=0\\ y_{z}\cos L+z_{z}\sin L=\frac{w_{z}}{w_{ie}} \end{array}$$

Solving Equation (15) yields two sets of solutions

(16) $$\{\begin{array}{l} x_{z}=-\frac{y_{y}y_{z}+z_{y}z_{z}}{x_{y}}\\ y_{z}=\frac{w_{z}}{w_{ie}\cos L}-z_{z}\tan L\\ z_{z}=\frac{-b\pm \sqrt{b^{2}-4ac}}{2a} \end{array}$$

where $\{\begin{array}{l} a=\frac{\left(x_{y}^{2}+y_{y}^{2}\right)\tan^{2}L-2y_{y}z_{y}\tan L+x_{y}^{2}+z_{y}^{2}}{x_{y}^{2}}\\ b=\frac{-2w_{z}\left(x_{y}^{2}+y_{y}^{2}\right)\tan L+2w_{z}y_{y}z_{y}}{w_{ie}x_{y}^{2}\cos L}\\ c=\frac{y_{y}^{2}w_{z}^{2}}{x_{y}^{2}w_{ie}^{2}\cos^{2}L}+\frac{w_{z}^{2}}{w_{ie}^{2}\cos^{2}L}-1 \end{array}$.

Then, $\vec{OA}$ can be obtained as shown in Equation (17)

(17) $$\vec{OA}=\vec{OB}\times \vec{OC}$$

Hence, from Equation (7), $C_{n}^{b}$ can be obtained. There are four sets of solutions for $C_{n}^{b}$ given $f_{y}$, $w_{y}$ and $w_{z}$.

Next, we consider the case where $f_{y}$, $w_{y}$ and $f_{x}$ are given. Likewise, given $f_{x}$ rather than $w_{z}$, we have

(18) $$z_{x}=\frac{f_{x}}{g}$$

Because $\vec{OA}$ is the unit vector of the positive $x_{b}$-axis and $\vec{OA}⊥\vec{OB}$, the following equations hold

(19) $$\{\begin{array}{l} \sqrt{x_{x}^{2}+y_{x}^{2}+z_{x}^{2}}=1\\ x_{y}x_{x}+y_{y}y_{x}+z_{y}z_{x}=0\\ z_{x}=\frac{f_{x}}{g} \end{array}$$

Solving Equation (19) yields two sets of solutions

(20) $$\{\begin{array}{l} x_{x}=\frac{-b\pm \sqrt{b^{2}-4ac}}{2a}\\ y_{x}=-\frac{x_{y}x_{x}+z_{y}z_{x}}{y_{y}}\\ z_{x}=\frac{f_{x}}{g} \end{array}$$

where $\{\begin{array}{l} a=x_{y}^{2}+y_{y}^{2}\\ b=2x_{y}z_{y}\frac{f_{x}}{g}\\ c=\left(y_{y}^{2}+z_{y}^{2}\right)\frac{f_{x}^{2}}{g^{2}}-y_{y}^{2} \end{array}$.

Therefore, $\vec{OC}$ is given by

(21) $$\vec{OC}=\vec{OA}\times \vec{OB}$$

Hence, $C_{n}^{b}$ can be obtained from Equation (7). There are four sets of solutions for $C_{n}^{b}$ given $f_{y}$, $w_{y}$, and $f_{x}$.

The coordinate transformation matrix $C_{n}^{b}$ has been derived in two typical cases. For both cases, one output of the three-axis accelerator and one output of the three-axis gyroscope, both in the same axis, should be selected. The case where the remaining measurement is another output of the three-axis gyroscope is denoted by FWW, e.g., $f_{y}$, $w_{y}$, and $w_{z}$, and the case where the remaining measurement is another output of the three-axis accelerator is denoted by FWF, e.g., $f_{y}$, $w_{y}$, and $f_{x}$.

There are six possible sets for FWW and six possible sets for FWF, as shown in Table 1.

**Table 1:** The six possible sets for FWW and FWF.

		1	2	3	4	5	6
	Output 1	$f_{x}$	$f_{x}$	$f_{y}$	$f_{y}$	$f_{z}$	$f_{z}$
	Output 2	$w_{x}$	$w_{x}$	$w_{y}$	$w_{y}$	$w_{z}$	$w_{z}$
FWW	Output 3	$w_{y}$	$w_{z}$	$w_{z}$	$w_{x}$	$w_{x}$	$w_{y}$
FWF	Output 3	$f_{y}$	$f_{z}$	$f_{z}$	$f_{x}$	$f_{x}$	$f_{y}$

For FWW, the three selected outputs are denoted as $f_{\alpha }$, $w_{\alpha }$ and $w_{\beta }$, where the subscripts α, β and γ represent x, y or z and should be different. The body frame is given by

(22) $$\{\begin{array}{l} x_{\alpha }=\pm \sqrt{1-y_{\alpha }^{2}-z_{\alpha }^{2}}\\ y_{\alpha }=\frac{w_{\alpha }}{w_{ie}\cos L}-z_{\alpha }\tan L\\ z_{\alpha }=\frac{f_{\alpha }}{g} \end{array}$$

(23) $$\{\begin{array}{l} x_{\beta }=-\frac{y_{\alpha }y_{\beta }+z_{\alpha }z_{\beta }}{x_{\alpha }}\\ y_{\beta }=\frac{w_{\beta }}{w_{ie}\cos L}-z_{\beta }\tan L\\ z_{\beta }=\frac{-b\pm \sqrt{b^{2}-4ac}}{2a} \end{array}$$

(24) $$\left[\begin{array}{c} x_{\gamma }\\ y_{\gamma }\\ z_{\gamma } \end{array}\right]=\{\begin{array}{c} \begin{array}{l} {\left[x_{\alpha },y_{\alpha },z_{\alpha }\right]}^{\text{T}}\times {\left[x_{\beta },y_{\beta },z_{\beta }\right]}^{\text{T}},\text{if}\;\text{M}\\ {\left[x_{\beta },y_{\beta },z_{\beta }\right]}^{\text{T}}\times {\left[x_{\alpha },y_{\alpha },z_{\alpha }\right]}^{\text{T}},\text{if}\;\text{N} \end{array} \end{array}$$

where *a*, *b* and *c* in Equation (23) are $\{\begin{array}{l} a=\frac{\left(x_{\alpha }^{2}+y_{\alpha }^{2}\right)\tan^{2}L-2y_{\alpha }z_{\alpha }\tan L+x_{\alpha }^{2}+z_{\alpha }^{2}}{x_{\alpha }^{2}}\\ b=\frac{-2w_{\beta }\left(x_{\alpha }^{2}+y_{\alpha }^{2}\right)\tan L+2w_{\beta }y_{\alpha }z_{\alpha }}{w_{ie}x_{\alpha }^{2}\cos L}\\ c=\frac{y_{\alpha }^{2}w_{\beta }^{2}}{x_{\alpha }^{2}w_{ie}^{2}\cos^{2}L}+\frac{w_{\beta }^{2}}{w_{ie}^{2}\cos^{2}L}-1 \end{array}$, M in Equation (24) denotes $\{\begin{array}{c} \alpha =x\\ \beta =y \end{array}$ or $\{\begin{array}{c} \alpha =y\\ \beta =z \end{array}$ or $\{\begin{array}{c} \alpha =z\\ \beta =x \end{array}$ and N in Equation (24) denotes $\{\begin{array}{c} \alpha =y\\ \beta =x \end{array}$ or $\{\begin{array}{c} \alpha =z\\ \beta =y \end{array}$ or $\{\begin{array}{c} \alpha =x\\ \beta =z \end{array}$.

For FWF, the three selected outputs are denoted as $f_{\alpha }$, $w_{\alpha }$ and $f_{\beta }$. The subscripts α, β and γ denote x, y or z and should be different. The body frame is given by Equations (22), (24), and (25):

(25) $$\{\begin{array}{l} x_{\beta }=\frac{-b\pm \sqrt{b^{2}-4ac}}{2a}\\ y_{\beta }=-\frac{x_{\alpha }x_{\beta }+z_{\alpha }z_{\beta }}{y_{\alpha }}\\ z_{\beta }=\frac{f_{\beta }}{g} \end{array}$$

where a, b, and c in Equation (25) are $\{\begin{array}{l} a=x_{\alpha }^{2}+y_{\alpha }^{2}\\ b=2x_{\alpha }z_{\alpha }\frac{f_{\beta }}{g}\\ c=\left(y_{\alpha }^{2}+z_{\alpha }^{2}\right)\frac{f_{\beta }^{2}}{g^{2}}-y_{\alpha }^{2} \end{array}$.

### 3.2. Choice of Solutions 

After ascertaining $O-x_{b}y_{b}z_{b}$, $C_{n}^{b}$ can be obtained from Equation (7). There are four possible solutions, but only one is appropriate. Given the four possible solutions of $C_{n}^{b}$, the vectors $f^{b}$ and $w^{b}$ can be computed from Equation (5). Comparing the calculated values of $f^{b}$ and $w^{b}$ with the original six outputs of the IMU, the appropriate solution can be easily ascertained amongst the four possible solutions.

To demonstrate the method for choosing the correct solution among the four possible solutions, one group of simulated outputs are used with L=32.026372°, $g=9.8{\text{ m/s}}^{2}$, and $w_{ie}=7.2921158\times 10^{-5}\text{ rad/s}$. $f_{y}$, $w_{y}$, and $w_{z}$ are selected to compute the attitude through the selective alignment method. Given the four possible solutions of the attitude (namely, $C_{n}^{b}$), the four possible sets of the six outputs can be computed from Equation (5), which are shown in Table 2.

**Table 2:** The four possible sets of six inertial measurement unit (IMU) outputs calculated with $f_{y}$, $w_{y}$, and $w_{z}$ used for selective alignment according to Equations (22)–(24).

	Angular Velocity ($10^{‒4}\mathrm{rad}/s$)			Specific Force ($m/s^{2}$)		
	$w_{x}$	$w_{y}$	$w_{z}$	$f_{x}$	$f_{y}$	$f_{z}$
1	−0.2550	0.6782	0.0824	−3.1497	3.3518	8.6536
2	0.2550	0.6782	0.0824	7.6192	3.3518	−5.1724
3	0.2550	0.6782	0.0824	3.1497	3.3518	8.6536
4	−0.2550	0.6782	0.0824	−7.6192	3.3518	−5.1724
Original outputs	−0.2550	0.6782	0.0824	−3.1497	3.3518	8.6536

It is easy to discern that the attitude for Set 1 in Table 2 is the correct solution by comparing the calculated and measured values of the outputs using one of the following sets of criteria:

- The signs of $f_{x}$ and $f_{z}$
- The signs of $w_{x}$ and $f_{z}$
- The approximate value of $f_{x}$.

The values compared will depend on the outputs selected. Through the selective alignment method, the attitude can be determined from any three outputs of the IMU in Table 1 in addition to at least one force component and one angular velocity component in the same axis. Some limited information from the remaining three outputs such as the signs and approximate value is also essential. Furthermore, if, for example, $f_{x}$, $f_{y}$, and $w_{z}$ are known, $f_{z}$ can be calculated from Equation (26). Then, the attitude by the selective alignment method can be obtained.

(26) $$\{\begin{array}{l} f_{x}^{2}+f_{y}^{2}+f_{z}^{2}=g^{2}\\ w_{x}^{2}+w_{y}^{2}+w_{z}^{2}=w_{ie}^{2} \end{array}$$

## 4. Exception in Selective Alignment

In practice, there are errors in the IMU outputs such as bias, and vibrations in the base will also affect the IMU outputs and thus the alignment. So, it is possible that $x_{\alpha }$ in Equation (22) will have an imaginary value, and thus there is no solution. Furthermore, there are singularities in Equations (23) and (25) when $x_{\alpha }$ and $y_{\alpha }$ equal zero, respectively. Thus, the problems are divided into two classes, those in which the denominator should be nonzero and those in which the radicand should be nonnegative. We consider the denominator problem first.

### 4.1. Nonzero Denominator

For FWW, if $x_{\alpha }$ equals zero, from Equation (15) we have

(27) $$\{\begin{array}{l} \sqrt{x_{\beta }^{2}+y_{\beta }^{2}+z_{\beta }^{2}}=1\\ y_{\alpha }y_{\beta }+z_{\alpha }z_{\beta }=0\\ y_{\beta }\cos L+z_{\beta }\sin L=\frac{w_{z}}{w_{ie}} \end{array}$$

Solving Equation (27) yields

(28) $$\{\begin{array}{l} x_{\beta }=\pm \sqrt{1-y_{\beta }^{2}-z_{\beta }^{2}}\\ y_{\beta }=\frac{w_{z}}{w_{ie}\cos L}-z_{\beta }\tan L\\ z_{\beta }=\frac{y_{\alpha }w_{\beta }}{w_{ie}(y_{\alpha }\sin L-z_{\alpha }\cos L)} \end{array}$$

In Equation (28), $\left(y_{\alpha }\sin L-z_{\alpha }\cos L\right)$ must be nonzero. If $(y_{\alpha }\sin L-z_{\alpha }\cos L)=0$, then because $x_{\alpha }=0$ we have ${\left[x_{\alpha },y_{\alpha },z_{\alpha }\right]}^{\text{T}}={\left[0,\cos L,\sin L\right]}^{\text{T}}$ or ${\left[x_{\alpha },y_{\alpha },z_{\alpha }\right]}^{\text{T}}={\left[0,-\cos L,-\sin L\right]}^{\text{T}}$, which indicates that the $\alpha_{b}$-axis coincides with $w_{ie}$. Thus, the β-axis should be in the plane that is perpendicular to $w_{ie}$. Because $w_{\beta }$ was also selected and must be zero, the β-axis must also be in the plane that is perpendicular to $w_{ie}$. Two coincident planes have an infinite number of lines of intersection, so only the $\alpha_{b}$-axis can be obtained in this particular case.

For FWF, if $y_{\alpha }$ is equal to zero, from Equation (19) we have

(29) $$\{\begin{array}{l} \sqrt{x_{\beta }^{2}+y_{\beta }^{2}+z_{\beta }^{2}}=1\\ x_{\alpha }x_{\beta }+z_{\alpha }z_{\beta }=0\\ z_{\beta }=\frac{f_{\beta }}{g} \end{array}$$

Solving Equation (29) yields

(30) $$\{\begin{array}{l} x_{\beta }=-\frac{z_{\alpha }z_{\beta }}{x_{\alpha }}\\ y_{\beta }=\pm \sqrt{1-x_{\beta }^{2}-z_{\beta }^{2}}\\ z_{\beta }=\frac{f_{\beta }}{g} \end{array}$$

In Equation (30), $x_{\alpha }$ should be non-zero. If $x_{\alpha }=0$, then we have ${\left[x_{\alpha },y_{\alpha },z_{\alpha }\right]}^{\text{T}}={\left[0,0,1\right]}^{\text{T}}$ or ${\left[x_{\alpha },y_{\alpha },z_{\alpha }\right]}^{\text{T}}={\left[0,0,-1\right]}^{\text{T}}$, which indicates that the α-axis coincides with g. Thus, the β-axis should be in the plane that is perpendicular to g. Because $f_{\beta }$ was selected and must be zero, the β-axis must also be in the plane that is perpendicular to g. Two coincident planes have an infinite number of lines of intersection, so only the $a_{b}$-axis can be obtained in this particular case.

### 4.2. Nonnegative Radicand 

To ensure that the value of $x_{\alpha }$ in Equation (22) is not imaginary, $\left(y_{\alpha }^{2}+z_{\alpha }^{2}\right)$ should be less than 1. However, it is possible that $f_{\alpha }$ can be slightly greater than g because of sensor errors or base vibrations. And, errors in $w_{\alpha }$ may also result in a negative radicand. Constructing algebraic rules to prevent a negative value of the radicand is complicated and abstract. Graphically, a negative value in the radicand occurs when the two conical surfaces in Figure 2a do not intersect or the plane and the conical surface in Figure 2b do not intersect. Therefore, negative values in the radicand can be avoided if these surfaces intersect.

**Figure 4:**
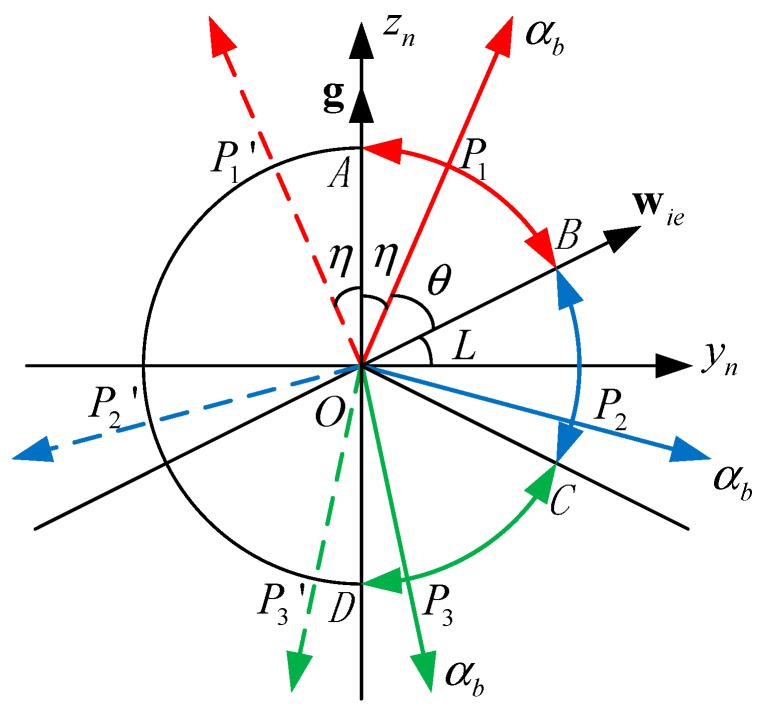
The critical point for the intersection of the two conical surfaces, where $P_{i}\prime$ is the reflection of $P_{i}$ about the $z_{n}$-axis and OC is the reflection of OB about the $y_{n}$-axis.

If the angle between the $\alpha_{b}$-axis and g is η, then $\cos\eta =\frac{f_{\alpha }}{g}$. Given $w_{\alpha }$ and denoting the angle between the $\alpha_{b}$-axis and $w_{ie}$ as θ, we have $\cos\theta =\frac{w_{\alpha }}{w_{ie}}$. Assuming η is accurate, θ must lie in a certain range that depends on η, or the two conical surfaces shown in Figure 2a cannot intersect. Referring to Figure 4, given η, let $∠AOP_{i}=\eta$. Then, θ must satisfy $∠BOP_{i}\leq \theta \leq ∠BOP_{i}\prime$, i=1,2,3. The range of θ based on η is given by

(31) $$\{\begin{array}{ll} \frac{\pi }{2}-L-\eta \leq \theta \leq \frac{\pi }{2}-L+\eta & 0\leq \eta <\frac{\pi }{2}-L\\ -\frac{\pi }{2}+L+\eta \leq \theta \leq \frac{\pi }{2}-L+\eta & \frac{\pi }{2}-L\leq \eta <\frac{\pi }{2}+L\\ -\frac{\pi }{2}+L+\eta \leq \theta \leq \frac{3\pi }{2}+L-\eta & \frac{\pi }{2}+L\leq \eta \leq \pi \end{array}$$

**Figure 5:**
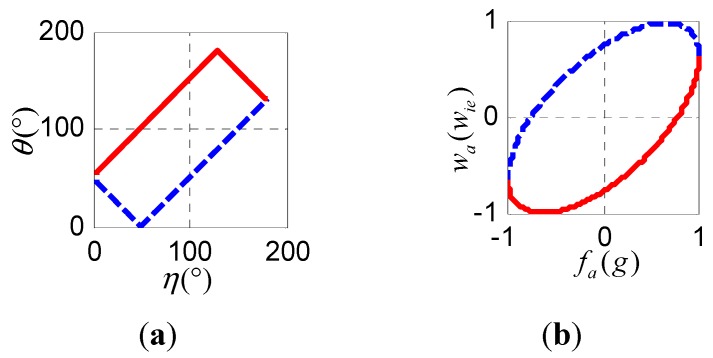
(**a**) The range of θ as a function of η, where the point (η,θ) must be within the rectangle; (**b**) The range of $w_{\alpha }$ as a function of $f_{\alpha }$, where the point $\left(f_{\alpha },w_{\alpha }\right)$ must be within the ellipse.

Figure 5a shows the range of θ for various values of η from 0 to π according to Equation (31).

$f_{\alpha }$ and $w_{\alpha }$ can be obtained through $f_{\alpha }=g\cos\eta$ and $w_{\alpha }=w_{ie}\cos\theta$. In this case, g and $w_{ie}$ can be considered constants. Given η and θ, we can plot the range of $w_{\alpha }$ as a function of $f_{\alpha }$. It follows that $\left(f_{\alpha },w_{\alpha }\right)$ must be within the ellipse shown in Figure 5b to ensure that the two conical surfaces in Figure 2a intersect and the radicand in Equation (22) will not be negative.

**Figure 6:**
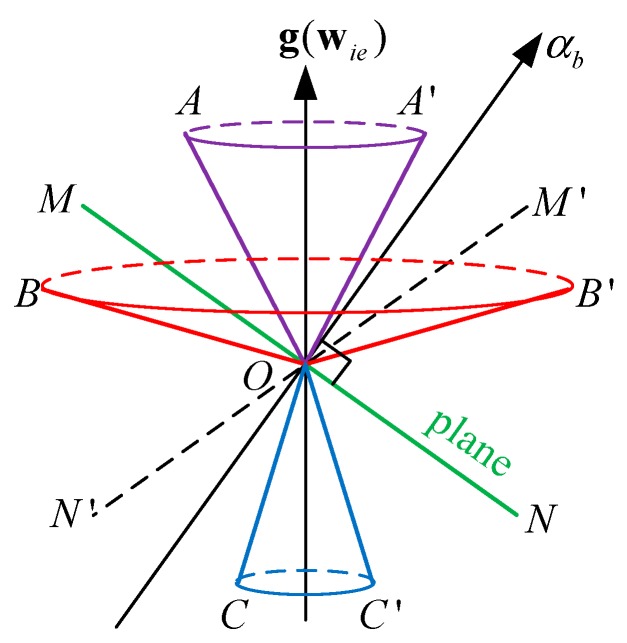
Intersection with the plane normal to the $\alpha_{b}$-axis and $g(w_{ie})$. The conical surfaces $AOA^{\prime }$, $BOB^{\prime }$, $COC^{\prime }$ are centered about $g(w_{ie})$ and the points $M^{\prime }$, $N^{\prime }$ are reflections of M, N, respectively, about $g(w_{ie})$.

After ascertaining the $\alpha_{b}$-axis, given another $w_{\beta }$ or $f_{\beta }$, the $\beta_{b}$-axis can be obtained. As shown in Figure 2b, the $\beta_{b}$-axis is formed by the intersection of the conical surface and the plane. As shown in Figure 6, MN is a plane normal to the $\alpha_{b}$-axis. The conical surfaces $AOA^{\prime }$ and $COC^{\prime }$ do not intersect the plane MN, but the conical surface $BOB^{\prime }$ does. It follows that only if B is in the area between OM and $ON^{\prime }$ will the conical surface $BOB^{\prime }$ intersect the MN plane. In other words, the conical surface $BOB^{\prime }$ is defined by $w_{\beta }$ or $f_{\beta }$, which should satisfy

(32) $$\{\begin{array}{l} \text{FWF}:f_{\alpha }^{2}+f_{\beta }^{2}\leq g^{2}\\ \text{FWW}:w_{\alpha }^{2}+w_{\beta }^{2}\leq w_{ie}^{2} \end{array}$$

If Equation (32) holds, the conical surface resulting from $f_{\beta }$ or $w_{\beta }$ intersects the plane normal to the $\alpha_{b}$-axis and the radicand in Equations (23) and (25) will not be negative.

#### 4.3. Exception Handling in the Algorithm

To ensure that the selective alignment method performs robustly, various exceptions must be addressed. In the first stage, where the α-axis is determined, if $\left|f_{\alpha }\right|$ is slightly greater than g because of bias in the accelerometer or base vibrations when the α-axis is nearly parallel to g, $f_{\alpha }$ should be substituted with $\frac{f_{\alpha }}{\left|f_{\alpha }\right|}g$. Similarly, if $\left|w_{\alpha }\right|$ is slightly greater than $w_{ie}$ because of drift in the gyroscope or base vibrations when the α-axis is nearly parallel to $w_{ie}$, $w_{\alpha }$ should be substituted with $\frac{w_{\alpha }}{\left|w_{\alpha }\right|}w_{ie}$. In addition, $\left(f_{\alpha },w_{\alpha }\right)$ should be within the area shown in Figure 5b; otherwise, $f_{\alpha }$ is substituted with $f_{\alpha }+\Delta f_{\alpha }$ or $w_{\alpha }$ is substituted with $w_{\alpha }+\Delta w_{\alpha }$ to make that $\left(f_{\alpha },w_{\alpha }\right)$ lie in the boundary of the ellipse shown in Figure 5b. In the second stage, where β-axis is obtained, $f_{\beta }$ or $w_{\beta }$ must satisfy Equation (32); if not, the algorithm substitutes for $f_{\beta }$ or $w_{\beta }$ so that the equality holds.

In practice, the algebraic operations may not result in values that are identically zero because of the limits of floating-point operations on the computer, so values within a certain tolerance around zero should be considered zero in the algorithm. We use $-10^{-12}<x<10^{-12}$ to judge if x is equal to zero.

Usually, four results can be obtained from the three selective outputs based on the selective alignment. There might be only two results or one result when there is only one intersection in Figure 2. If no intersection occurs because of bias errors or vibration, the value of the relevant selected output is changed to ensure an intersection. Because all six original outputs are given, the correct value of the attitude is picked out by minimizing the following cost function for ease of computer programming, although two signs or one approximate value from the remaining outputs could be enough to obtain the right result, as explained in Section 3.2.

**Figure 7:**
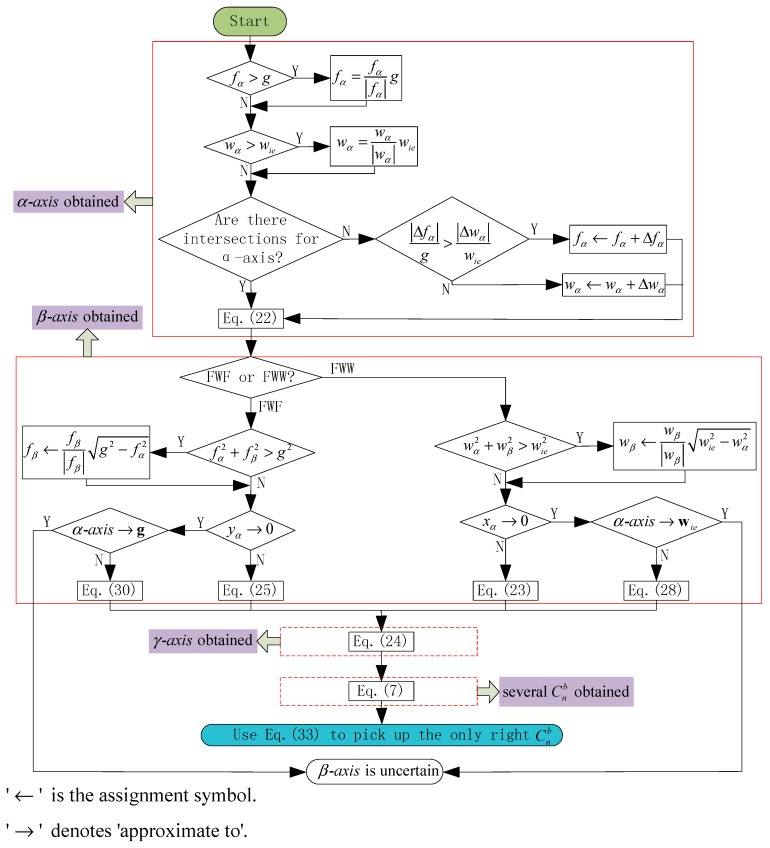
Flowchart for the selective alignment algorithm.

(33) $$\delta =\left|\frac{w^{b}-C_{n}^{b}w_{ie}^{n}}{w_{ie}}\right|+\left|\frac{f^{b}-C_{n}^{b}g^{n}}{g}\right|$$

where $w^{b}$ and $f^{b}$ are the original outputs of the IMU, $C_{n}^{b}$ is computed from Equation (7), which may have four different values, and |⋅| is the norm operator.

To handle exceptions, a binary number with five bits, $b_{5}b_{4}b_{3}b_{2}b_{1}$, is used. If there are no exceptions, $b_{5}b_{4}b_{3}b_{2}b_{1}=00000$; otherwise,

- $b_{1}=1$ if $f_{\alpha }>g$
- $b_{2}=1$ if $w_{\alpha }>w_{ie}$
- $b_{3}=1$ if there is no intersection with the $\alpha_{b}$-axis
- $b_{4}=1$ if in FWW, $w_{\alpha }^{2}+w_{\beta }^{2}>w_{ie}^{2}$, or if in FWF, $f_{\alpha }^{2}+f_{\beta }^{2}>g^{2}$
- $b_{5}=1$ if in FWW, $\alpha_{b}$-axis$\to w_{ie}$, or if in FWF, $\alpha_{b}$-axis→g (The symbol “→” denotes “approximate to”)

The exceptions rarely happen unless the SINS is level (one of the body axes is along the direction of the gravity) or one of the body axes is parallel to the axis of rotation of the Earth. If $b_{5}=1$, the attitude cannot be obtained. The other four exceptions arise because of measurement errors or base vibrations, which do not significantly affect the selective alignment after corresponding exception handling. Figure 7 shows a flowchart for the selective alignment algorithm.

## 5. Simulations and Experiments

Simulations and experiments were conducted to validate the performance of the proposed selective alignment method.

### 5.1. Static Alignment Example 

Similar to the coordinate transformation matrix $C_{n}^{b}$, the Euler angles, *i.e.*, roll, pitch, and yaw, can also be used to express the attitude [4]. 

Simulations were conducted to test the selective alignment method. Because the alignment is usually performed in a short period of time, only the bias and noise in the IMU were considered. With the SINS remaining static on the ground, the AA methods including the selective alignment method and the analytic coarse alignment method were used to obtain the attitude angles. The values of the various parameters were chosen as follows:
Simulation conditions for the static alignment:
L=32°, $g=9.8m/s^{2}$, $w_{ie}=7.2921158\times 10^{‒5}\mathrm{rad}/s$, ${\left[\text{roll},\text{pitch},\text{yaw}\right]}^{\text{T}}={\left[10{}^{\circ},10{}^{\circ},10{}^{\circ}\right]}^{\text{T}}$,
${\left[\nabla_{x},\nabla_{y},\nabla_{z}\right]}^{\text{T}}={\left[10^{-4},10^{-4},10^{-4}\right]}^{\text{T}}\times g$ , ${\left[\varepsilon_{x},\varepsilon_{y},\varepsilon_{z}\right]}^{\text{T}}={\left[0.01,0.01,0.01\right]}^{\text{T}}\;{}^{\circ}\text{/h}$,${\left[\sigma_{f_{x}},\sigma_{f_{y}},\sigma_{f_{y}}\right]}^{\text{T}}={\left[5\times 10^{-5},5\times 10^{-5},5\times 10^{-5}\right]}^{\text{T}}\times g$ , ${\left[\sigma_{w_{x}},\sigma_{w_{y}},\sigma_{w_{z}}\right]}^{\text{T}}={\left[0.005,0.005,0.005\right]}^{\text{T}}\;{}^{\circ}\text{/h}$
where $\nabla_{i}$ is the bias for the i-axis accelerometer, $\varepsilon_{i}$ is the drift (assumed constant) for the i-axis gyroscope, $\sigma_{f_{i}}$ is the standard deviation of the white noise for the i-axis accelerometer, and $\sigma_{w_{i}}$ is the standard deviation of the white noise for the i -axis gyroscope. The sampling rate was 100 Hz, and the IMU outputs were sampled for one minute. The average values were computed and used to test the AA method. There were twelve sets as a result of the various possibilities in the selective alignment method. The alignment results are shown in Table 3.

**Table 3:** The attitude angles obtained using analytic alignment (AA) methods in the simulation.

	$f_{x}$ $w_{x}$ $f_{y}$ $w_{y}$ $f_{z}$ $w_{z}$						Roll (°)	Pitch (°)	Yaw (°)	$b_{5}b_{4}b_{3}b_{2}b_{1}$
**Selective Alignment**							9.9943	10.0058	9.9572	00000
							9.9970	10.0910	9.9725	00000
							9.9933	9.9732	9.9514	00000
							9.9931	9.9664	9.9502	00000
							9.9943	10.0058	9.7485	00000
							10.4947	10.0058	9.7485	00000
							9.9606	10.0058	9.7485	00000
							9.9386	10.0058	9.7485	00000
							9.9933	9.9732	9.8989	00000
							10.0015	9.9649	9.9464	00000
							9.9606	10.0058	9.7106	00000
							9.9651	10.0013	9.7365	00000
**Analytic Coarse Alignment**	Equation (6a)						9.9933	10.0058	9.9451	-
	Equation (6b)						9.9933	10.0058	9.9445	-
	True attitude						10	10	10	-

Where the contents from left to right in the six grids denote $f_{x}$, $w_{x}$, $f_{y}$, $w_{y}$, $f_{z}$, and $w_{z}$, respectively. The corresponding contents are selected when being painted.

Next, assume ${\left[\text{roll},\text{pitch},\text{yaw}\right]}^{\text{T}}={\left[0{}^{\circ},0{}^{\circ},0{}^{\circ}\right]}^{\text{T}}$, which is more likely to occur in practice, and the values of the other parameters were left unchanged. The results are shown in Table 4.

**Table 4:** The attitude angles obtained using AA methods in the simulation.

	$f_{x}$ $w_{x}$ $f_{y}$ $w_{y}$ $f_{z}$ $w_{z}$						Roll (°)	Pitch (°)	Yaw (°)	$b_{5}b_{4}b_{3}b_{2}b_{1}$
**Selective Alignment**							−0.0058	0.0058	359.9588	00000
							−0.0058	0.0715	359.9588	00000
							−0.0058	0	359.9588	01000
							−0.0058	−0.0451	359.9588	00000
							−0.0058	0.0058	0	00100
							−0.0717	0.0058	0	00100
							0	0.0058	0	01100
							0	0.0058	0	01100
							−0.0058	−0.0447	7.3397	10101
							0	−0.0451	359.9552	00101
							0.0447	0.0058	262.5998	10101
							0	−0.0451	0	01101
**Analytic Coarse Alignment**	Equation (6a)						−0.0058	0.0058	359.9588	-
	Equation (6b)						−0.0058	0.0058	359.9588	-
	True attitude						0	0	0	-

From Table 4, it can be observed that intolerant errors of the yaw angle occur due to the $b_{5}=1$ exception in $\left(f_{z},w_{z},f_{x}\right)$ and $\left(f_{z},w_{z},f_{y}\right)$ for the selective alignment because they are quite close to the extreme state $\alpha_{b}$-axis→g in the FWF mode where the β−axis is uncertain when the SINS is level, as explained in Section 4.1. The other exceptions except the $b_{5}=1$ exception do not significantly affect the selective alignment. From Table 3, no exception occurs, which indicates that the exceptions rarely happen unless the SINS is level or one of the body axes is parallel to the axis of rotation of the Earth. In general, the selective alignment and the analytic coarse alignment could achieve consistent alignment accuracy, and the alignment accuracy would be decreased when two gyroscope outputs are selected for the selective alignment.

### 5.2. Fault Detection Example 

The selective alignment method may be potentially an effective approach for sensor fault detection such as that prior to take-off in aircraft. That is, by determining the alignment with independent sensors, individual sensor failures could be identified on the ground. A simulation was performed with self-alignment and assuming a 0.1%-level IMU. The accuracy of the z-axis gyroscope was assumed to be degraded.

Simulation conditions for fault detection:${\left[\nabla_{x},\nabla_{y},\nabla_{z}\right]}^{\text{T}}={\left[10^{-4},10^{-4},10^{-4}\right]}^{\text{T}}\times g$, ${\left[\varepsilon_{x},\varepsilon_{y},\varepsilon_{z}\right]}^{\text{T}}={\left[0.01,0.01,1\right]}^{\text{T}}\;{}^{\circ}\text{/h}$,${\left[\sigma_{f_{x}},\sigma_{f_{y}},\sigma_{f_{y}}\right]}^{\text{T}}={\left[5\times 10^{-5},5\times 10^{-5},5\times 10^{-5}\right]}^{\text{T}}\times g$, ${\left[\sigma_{w_{x}},\sigma_{w_{y}},\sigma_{w_{z}}\right]}^{\text{T}}={\left[0.005,0.005,0.005\right]}^{\text{T}}\;{}^{\circ}\text{/h}$,${\left[\text{roll},\text{pitch},\text{yaw}\right]}^{\text{T}}={\left[0{}^{\circ},0{}^{\circ},30{}^{\circ}\right]}^{\text{T}}$

**Figure 8:**
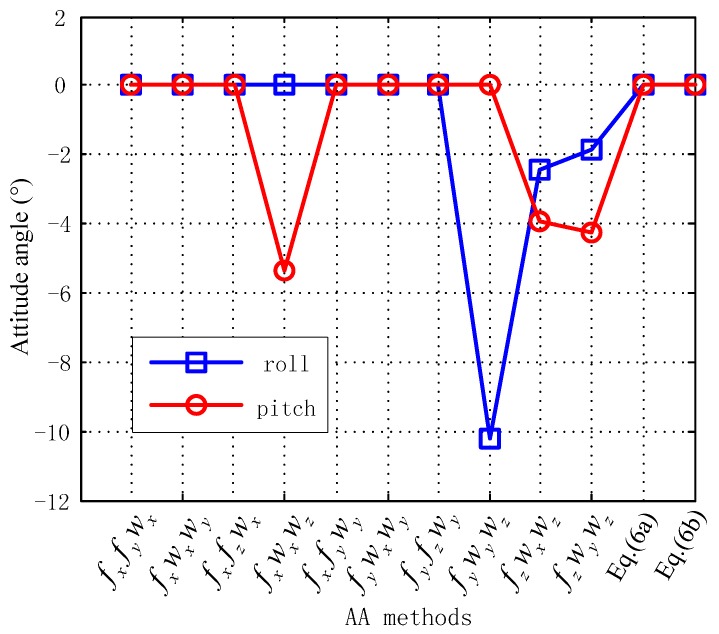
The alignment results using selective alignment for fault detection.

The IMU outputs were sampled for ten seconds. The average values were computed, and these are denoted as $w^{b}$ and $f^{b}$. The difference between the norm of $w^{b}$ and $w_{ie}$ was 0.556 °/h, and the difference between the norm of $f^{b}$ and g was $1.0121\times 10^{-4}g$. It is obvious that several of the outputs of the three-axis gyroscope are faulty. The selective alignment method can be further used to identify the erroneous output. The alignment results are shown in Figure 8. Because the platform was level, the two sets of outputs $\left(f_{z},w_{z},f_{x}\right)$ and $\left(f_{z},w_{z},f_{y}\right)$ were not selected for use in the selective alignment method in case of the $b_{5}=1$ exception.

Because the outputs of the three-axis accelerometer are correct, the roll and pitch angles obtained through the analytic coarse alignment method are rational. From Figure 8, once $w_{z}$ is selected for the selective alignment method, the roll angle or the pitch angle will have obvious errors. It can be observed that the z-axis output of the three-axis gyroscope is severely affected.

### 5.3. Self-Calibration Example

An error in the z-axis gyroscope will cause errors mainly in the yaw angle in the inertial navigation process. To reach a balance between cost and accuracy, an IMU may include one high-accuracy z-axis gyroscope and two other less accurate gyroscopes. Using the selective alignment method with the outputs of the higher-accuracy z-axis gyroscope and the accelerometers, the attitude can be obtained. Then, the errors in the other, lower-accuracy gyroscopes can be compensated.

A simulation was performed to test the validity of the self-calibration procedure. The SINS was assumed to be static on the ground, and the SINS was assumed not to be level to avoid the $b_{5}=1$ exception.

Simulation conditions for the self-calibration:${\left[\nabla_{x},\nabla_{y},\nabla_{z}\right]}^{\text{T}}={\left[10^{-4},10^{-4},10^{-4}\right]}^{\text{T}}\times g$, ${\left[\varepsilon_{x},\varepsilon_{y},\varepsilon_{z}\right]}^{\text{T}}={\left[0.01,0.01,1\right]}^{\text{T}}\;{}^{\circ}\text{/h}$,
${\left[\sigma_{f_{x}},\sigma_{f_{y}},\sigma_{f_{y}}\right]}^{\text{T}}={\left[5\times 10^{-5},5\times 10^{-5},5\times 10^{-5}\right]}^{\text{T}}\times g$, ${\left[\sigma_{w_{x}},\sigma_{w_{y}},\sigma_{w_{z}}\right]}^{\text{T}}={\left[0.05,0.05,0.005\right]}^{\text{T}}\;{}^{\circ}\text{/h}$,${\left[\text{roll},\text{pitch},\text{yaw}\right]}^{\text{T}}={\left[20{}^{\circ},20{}^{\circ},20{}^{\circ}\right]}^{\text{T}}$

**Table 5:** The AA results for the self-calibration test.

	$f_{x}$ $w_{x}$ $f_{y}$ $w_{y}$ $f_{z}$ $w_{z}$						Roll (°)	Pitch (°)	Yaw (°)
**Selective Alignment**							19.9943	20.0061	19.5203
							20.3557	22.5326	20.5163
							19.9922	19.9900	19.5140
							20.0003	20.0516	19.5379
							19.9943	20.0061	18.5666
							27.9823	20.0061	18.5666
							19.9760	20.0061	18.5666
							20.6650	20.0061	18.5666
							19.9922	19.9900	19.8515
							19.8948	20.0868	19.5674
							19.9760	20.0061	19.8044
							19.6457	20.3300	18.8464
**Analytic Coarse Alignment**	Equation (6a)						19.9922	20.0061	19.4363
	Equation (6b)						19.9922	20.0061	19.4388
	True attitude						20	20	20

The IMU outputs were sampled for one minute. The average values were computed and then used for the compensation. The alignment results are given in Table 5. It can be observed that the attitude for the selective alignment method using $\left(f_{z},w_{z},f_{x}\right)$ produced the best result. Having computed $C_{n}^{b}$ through the $\left(f_{z},w_{z},f_{x}\right)$ option of selective alignment, the constant drifts of the two lower-accuracy gyroscopes were obtained from Equation (34). The drift rates were ${\hat{\varepsilon }}_{x}=0.0694\;{}^{\circ}\text{/h}$ and ${\hat{\varepsilon }}_{y}=0.0894\;{}^{\circ}\text{/h}$, and the drift errors of the two lower-accuracy gyroscopes are well compensated without multi-position.

(34) $$\hat{\varepsilon }=w_{b}-C_{n}^{b}w_{ie}^{n}$$

### 5.4. Alignment in the Static Vehicle 

**Figure 9:**
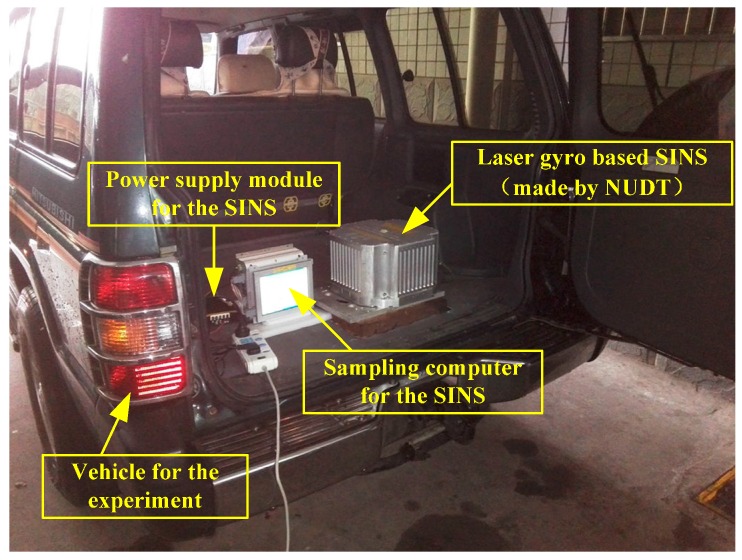
The vehicular experiment scene and the related equipment.

The experiment was performed at the National University of Defense Technology (NUDT). The SINS used in the tests, shown in Figure 9, was built by NUDT and includes three 90-type mechanically dithered ring laser gyroscopes. The accuracy of the gyroscope is 0.01 °/h, and the accuracy of the accelerometer is 100 ug.

**Figure 10:**
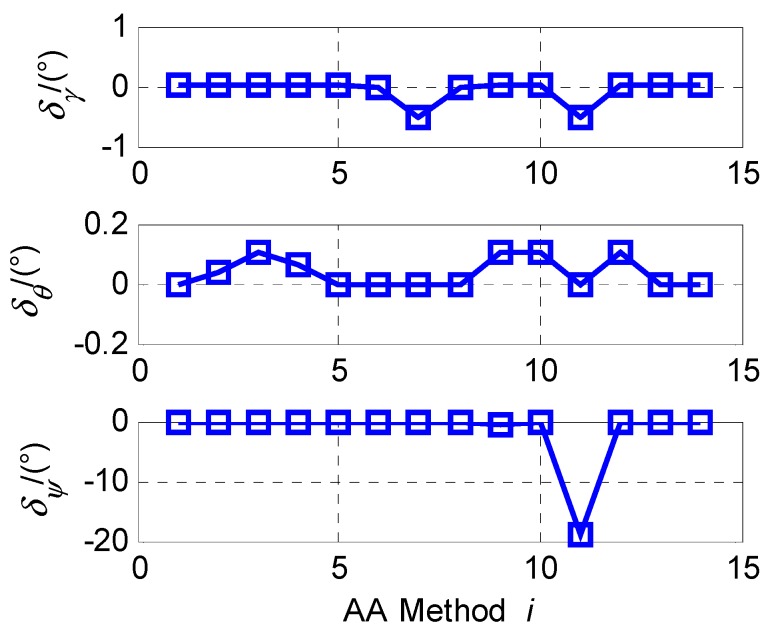
The errors of the attitude obtained using AA methods.

First, the vehicle remained static and the engine was not working. The outputs of the IMU were sampled for ten minutes. The average of the last two minutes’ data was computed and then used for the AA. The alignment results using fine alignment method given the data of ten minutes were considered as the true attitude. The alignment results are shown in Table 6. The errors of the attitude for the 14 AA methods are shown in Figure 10, where $\delta_{\gamma }$, $\delta_{\theta }$ and $\delta_{\psi }$ denote the errors of the roll angle, pitch angle and yaw angle, respectively.

From Table 6 and Figure 10, when $\left(f_{x},f_{z},w_{z}\right)$ or $\left(f_{y},f_{z},w_{z}\right)$ are selected to conduct the selective alignment. The error of the yaw angle is worse. That is because they are close to the $b_{5}=1$ exception as described in Section 4.3. Besides, if $f_{y}$ is selected for the selective alignment, the pitch angles are the same. If the three selections from the six IMU outputs for the selective alignment include two gyroscope outputs or exclude $f_{x}$ or $f_{y}$, the accuracy of the roll or pitch is worse, as shown in Table 6.

**Table 6:** The attitude angles obtained using AA methods.

		$f_{x}$ $w_{x}$ $f_{y}$ $w_{y}$ $f_{z}$ $w_{z}$						Roll (°)	Pitch (°)	Yaw (°)
**Selective Alignment**	1							−0.0744	1.6172	292.8975
	2							−0.0744	1.6537	292.8974
	3							−0.0744	1.7247	292.8973
	4							−0.0744	1.6760	292.8974
	5							−0.0744	1.6172	292.9183
	6							−0.0899	1.6172	292.9183
	7							−0.6041	1.6172	292.9183
	8							−0.0997	1.6172	292.9183
	9							−0.0744	1.7247	292.1757
	10							−0.0536	1.7255	292.8692
	11							−0.6041	1.6172	274.1607
	12							−0.0539	1.7255	292.8565
**Analytic Coarse Alignment**	13	Equation (6a)						−0.0744	1.6172	292.9162
	14	Equation (6b)						−0.0744	1.6172	292.9145
		True attitude						−0.0745	1.6173	292.9206

### 5.5. Alignment in the Vehicle with Vibration 

Because vibration is common in actual systems, the alignment in the base with vibration is studied. The vehicle remained static for ten minutes, then fire the engine and let the engine idle. The outputs of the three-axis gyroscope were greatly affected because of vibration caused by the idle engine. And the yaw angle obtained through AA could be greatly influenced. The outputs from the IMU for two minutes during the time when the engine was idling were used for the application of the AA. And the alignment results using fine alignment method given the outputs of the whole time were considered as the true attitude.

Since the six outputs from the IMU have a redundancy for the AA. The accuracy of the alignment could be increased compared to other AA methods by using two or more sets of selections for the selective alignment and grouping the alignment results in a certain way. The roll and pitch angles can be determined due to the outputs of the three-axis accelerometer, while outputs from the three-axis gyroscope are required to obtain the yaw angle. The outputs of the three-axis gyroscope are much more easily influenced than the three-axis accelerometer in the base with vibration. The following AA method based on the selective alignment is presented. The yaw angle obtained through the selective alignment with the selection of $\left(f_{x},f_{y},w_{x}\right)$ is denoted by $\psi_{x}=h_{x}\left(w_{x}\right)$. The yaw angle obtained through the selective alignment with the selection of $\left(f_{x},f_{y},w_{y}\right)$ is denoted by $\psi_{y}=h_{y}\left(w_{y}\right)$. Approximatively, $w_{x}\sim N\left(u_{x},\sigma_{x}^{2}\right)$, $w_{y}\sim N\left(u_{y},\sigma_{y}^{2}\right)$, where $N\left(u_{t},\sigma_{t}^{2}\right)$ denotes the normal distribution with the mean $u_{t}$ and the standard deviation $\sigma_{t}$, t=x,y. $u_{t}$ was estimated as the average value of the data for two minutes from the output in t-axis gyroscope, and $\sigma_{t}^{2}$ was estimated as the computed variance value from the same data divided by the number of the samples. The variance of the yaw angle could be decreased using

(35) $$\psi_{xy}=\left(1-k\right)\psi_{x}+k\psi_{y}$$

where $k=\frac{D\left(\psi_{x}\right)}{D\left(\psi_{x}\right)+D\left(\psi_{y}\right)}$, and it results in $D\left(\psi_{xy}\right)=\frac{D\left(\psi_{x}\right)D\left(\psi_{y}\right)}{D\left(\psi_{x}\right)+D\left(\psi_{y}\right)}$. D(⋅) is the variance operator.

$D\left(\psi_{i}\right)$ was computed using a simple method by constructing three-element array. The principle of the method is described as follows. If $w\sim N\left(u,\sigma^{2}\right)$, then

(36) $$E\left(w\right)=u,\;D\left(w\right)=\sigma^{2},\;E\left(w^{2}\right)=u^{2}+\sigma^{2},\;E\left(w^{3}\right)=u^{3}+3u\sigma^{2}$$

where E(⋅) is the expect operator. Construct $w={\left[u-\sqrt{\frac{3}{2}}\sigma ,u,u+\sqrt{\frac{3}{2}}\sigma \right]}^{\text{T}}$, which satisfies

(37) $$E\left(w\right)=u,\;D\left(w\right)=\sigma^{2},\;E\left(w^{2}\right)=u^{2}+\sigma^{2},\;E\left(w^{3}\right)=u^{3}+3u\sigma^{2}$$

It can be observed that the estimation accuracy using the three-element array can reach the third order.

Similarly, in order to compute the variance of the yaw angle due to the influenced gyroscope output, construct $w_{t}={\left[u_{t}-\sqrt{\frac{3}{2}}\sigma_{t},u_{t},u_{t}+\sqrt{\frac{3}{2}}\sigma_{t}\right]}^{\text{T}}$as the outputs from one-axis gyroscope, the variance of the yaw angle using the selective alignment method can be estimated by

(38) $$D\left(\psi_{t}\right)=\frac{1}{3}\sum_{i=1}^{3}\left\{h_{t}\left[w_{t}\left(i\right)\right]-\bar{\psi_{t}}\right\}$$

where $\bar{\psi_{t}}=\frac{1}{3}\sum_{i=1}^{3}h_{t}\left[w_{t}\left(i\right)\right]$, t=x,y.

**Figure 11:**
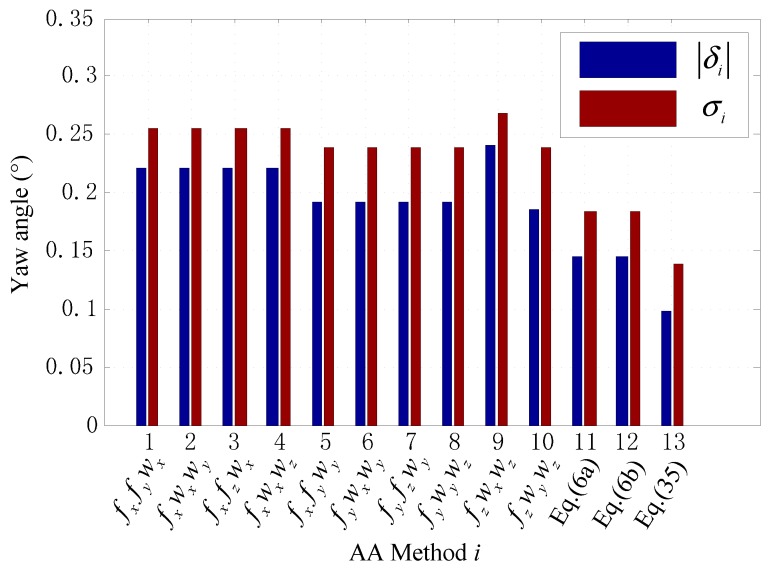
The errors of the yaw angle obtained using AA methods.

The experiment was conducted for fifty times. Assume the yaw angle using the i-th method shown in Figure 11 for the j-th experiment is ${\hat{\psi }}_{ij}$, and the corresponding true value using fine alignment method is $\psi_{j}$. $\left|\delta_{i}\right|$ and $\sigma_{i}$ are used to test the average alignment error and the stability, respectively, for the i-th method shown in Figure 11. They are given by

(39) $$\left|\delta_{i}\right|=\frac{1}{50}\sum_{j=1}^{50}\left|{\tilde{\psi }}_{ij}\right|,\;\sigma_{i}=\sqrt{\frac{1}{50-1}\sum_{j=1}^{50}{\left({\tilde{\psi }}_{ij}-{\bar{\tilde{\psi }}}_{i}\right)}^{2}}$$

where ${\tilde{\psi }}_{ij}={\hat{\psi }}_{ij}-\psi_{j}$, ${\bar{\tilde{\psi }}}_{i}=\frac{1}{50}\sum_{j=1}^{50}{\tilde{\psi }}_{ij}$.

The results are shown in Figure 11. It can be observed that the yaw angle using Equation (35) based on the selective alignment achieves the best accuracy and stability among the AA methods. However, it has to be noted that AA could usually not achieve better alignment result in the base with obvious vibration than INCA presented in the introduction section. Nevertheless, AA has its vitality for the self-alignment of the SINS on the ground, especially in the base with limited vibration, where both AA and INCA could achieve consistent alignment precision and AA requires less alignment time and has much lower computational complexity. Moreover, the principle of the selective alignment shows that two vectors are redundant to determine the coordinate transformation matrix, while at least two vectors are required for the TRIAD algorithm. Given the idea, some more improvement may be achieved in some other applications where TRIAD algorithm is used such as the spacecraft attitude determination problem.

## 6. Conclusions

A new AA method called selective alignment is proposed, which uses only three outputs of the IMU and a few properties from the remaining outputs such as the sign and the approximate value to obtain the attitude of the SINS relative to the Earth in the static base on the ground. Simulations and experiments were conducted, and the results demonstrate that the selective alignment can be used for self-alignment, fault detection and self-calibration, and the selective alignment could achieve better alignment result than the analytic coarse alignment which is based on the TRIAD algorithm. Selective alignment algorithm, as an extension of the TRIAD algorithm, implies that two non-coplanar vectors are redundant for determining the transformation matrix, which could be studied further in other attitude determination problems where TRIAD algorithm is applied such as spacecraft attitude estimation.
